# Functionalized microneedles for continuous glucose monitoring

**DOI:** 10.1186/s40580-018-0161-2

**Published:** 2018-10-24

**Authors:** Kai Takeuchi, Beomjoon Kim

**Affiliations:** Institute of Industrial Science, The University of Tokyo, 4-6-1 Komaba, Meguro-ku, Tokyo, 153-8505 Japan

**Keywords:** Microneedles, Glucose sensor, Interstitial fluids

## Abstract

Microneedles (MNs) have been established as promising medical devices as they are minimally invasive, cause less pain, and can be utilized for self-administration of drugs by patients. There has been rapid development in MNs for transdermal monitoring and diagnostic systems, following the active research on fabrication methods and applications for drug delivery. In this paper, recent investigations on bio-sensing using MNs are reviewed in terms of the applicability to continuous glucose monitoring system (CGMS), which is one of the main research focuses of medical engineering technologies. The trend of the functionalized MNs can be categorized as follows: (i) as a sensing probe, and (ii) as a biological fluid collector. MNs as in vivo sensors are mainly integrated or coated with conductive materials to have the function as electrodes. MNs as fluid collectors are given a certain geometrical design, such as a hollow and porous structure aided by a capillary action or negative pressure, to extract the interstitial fluids or blood for ex vivo analysis. For realization of CGMS with MNs, a long-term accurate measurement by the MN-based sensing probe or a fluidic connection between the MN-based fluid collector and the existing microfluidic measurement systems should be investigated.

## Introduction

Diagnostic systems using bio-sensing technologies have been the main focus of research for the last few decades owing to the high demand for more rapid, precise, and easy-to-use applications for the well-being of society. One of the aims in the development of recent diagnostic devices is to achieve a shorter time period of analysis with a reduced amount of the required biological samples by microfluidic and electrochemical components realized by new fabrication technologies, such as the CMOS and MEMS. However, in spite of the advantages of such diagnostic systems, called micro total analysis systems (pTASs), the methodologies of body fluid sampling is limited to conventional methods such as hypodermic needles.

Conventional needles have several disadvantages such as the associated unavoidable pain, and requirement of a high level of skilled training of the medical professionals, even though they extract too much amount of the body fluids for pTASs. Hence, microneedles (MNs) have attracted significant attention as next-generation devices to access biological tissues through the skin without pain due to their microsized dimension, e.g. typically a length of less 1 mm and diameter of less 500 pm. Subsequent to the reports of MNs being novel tools to penetrate the skin and to deliver drugs and vaccines with a minimal invasion and pain, a wide range of research has focused on the methods of their fabrication, supported by micromachining technologies and the applications for transdermal, self-administration of drugs [1–12].

In addition to the medical drug delivery, MNs, e.g., dissolvable MN patches, are also well known as commercialized cosmetic products for skin treatments [13, 14] nowadays, enabling an efficient localized drug delivery process, self-administered by the patient, with less fear of skin penetration [15].

Of late, MNs are expected to be applied to the extraction of biological information out of human skin in contrast to drug delivery into the skin through micro-sized pores. The targets of MN bio-sensing are blood or interstitial fluids (ISFs), as the MNs can reach a depth of approximately 1 mm where the vessels are located and ISF surrounds the cells in the epidermis. The analysis methodologies of such biological fluids are well developed with the advancement of both enzymatic and non-enzymatic electrochemical science for the detection and measurement of medical biomarkers such as metabolites, ions, proteins, and glucose. In the field of MN-based diagnostic systems, ISF is the main target sample since ISF contains various valuable biomarkers such as K^+^ and Na^+^ ions, nitrogen oxide, and glucose, the analysis, as well as that of blood [16].

 In particular, the glucose measurement is the focus of research on the bio-sensing in accordance with societal demands, as Type 1 and 2 diabetes are, nowadays, one of the most serious diseases due to its long-term effects on the health of patients, the large number of patients involved, and the huge amount of social and economical expenses. Currently, the blood glucose level is monitored by self-monitoring blood glucose (SMBG) devices in which the patients are required to prick their fingers to extract a drop of blood 2–5 times a day for colorimetric or enzymatic electrochemical measurements, which results in a physical and mental strain on the patients [17].

Furthermore, the concept of continuous glucose monitoring system (CGMS) has been initiated as a more efficient methodology to control the blood glucose level for prevention, diagnosis, and treatment of diabetes. CGMS allows a seamless observation of a change in the blood glucose level, even when the patients are not able to measure the glucose level by themselves [18–21]. However, the process of monitoring is carried out by conventional needles that restrict the activities of the patients, as certain movements of the body may cause some pain or dislodge the implanted needle.

For these reasons, monitoring of the blood glucose level is the main objective of research in MN-based bio-sensing, due to its applicability to point-of-care or continuous diagnostic devices without pain. Even though ISF reflects the change in the blood glucose level after a 4–10 min delay [22], it is still the target of the MN-based glucose sensors, so as to avoid significant pain by the penetration. The basic strategy of the MN-based glucose sensor is to utilize the amperometric measurement with immobilized glucose oxidase (GO_x_) for the detection of H_2_O_2_ generated by the reaction given below:

1 $${\text{Glucose }} + {\text{O}}_{2} \,  {\xrightarrow{\text{GOx}}} \, {\text{ Gluconic acid }} + {\text{ H}}_{ 2} {\text{O}}_{ 2} .$$

The generated H_2_O_2_ is detected by a working electrode (WE), following the re-action given below [23]:

2 $${\text{H}}_{ 2} {\text{O}}_{ 2} \, {\xrightarrow{\text{+700mV}}} \, {\text{O}}_{ 2} + {\text{ 2H}}^{+} + {\text{ 2e}}^{-}.$$

In addition to the amperometric measurement, potentiometric and optical methods such as enzyme field effect transistor (FET)-based [24, 25], ion selective field effect transistor (ISFET)-based [26, 27], and infrared (IR) light-based measurements [28, 29] have been widely explored. Thus, in order to develop clinically diagnostic systems using MNs, the MNs should have specific functions according to those methodologies, in addition to their own capability of penetrating the skin.

On the basis of this concept, MNs have been integrated with a variety of measurement systems by a specialized design of the material of MN, its configuration, and structure. These studies are emerging rapidly from the level of laboratory to clinical tests. Therefore, even though there are excellent review papers already available on bio-sensing using MNs [30–32], it is necessary to summarize the present-day investigations to have a full picture of the research conducted so far on MN-based glucose sensors, especially CGMS.

In this paper, a review of the recent progress on the MN devices functionalized for diagnostic systems, especially glucose monitoring, is presented. The functionally developed MNs for diagnostic bio-sensing are categorized into MNs as (i) sensing probes and (ii) biological fluids collectors, according to their applications. Those functionalized MNs are discussed from the perspective of the applicability for CGMS devices.

## MNs as sensing probes

### MNs coated with conductive materials

Miniaturization of a sensing probe to form an MN configuration is a natural way for minimally invasive medical monitoring, as successful results on the detection and measurement of biomarkers such as glucose were achieved by using implantable biosensors [21, 33]. As the scientific methodology for the measurement of analytes in ISF using electrochemical components has been highly developed, it is justifiable to functionalize MNs as in vivo sensing probes beneath the skin, enabling point-of-care measurement owing to their easy penetration into the skin. For this, MNs should be combined with conductive materials as electrodes to connect electrochemical reactions in the body and external amperometric or potentiometric measurement systems.

One of the methods to provide MNs electric function is to coat the MN with electrochemical components. As a result of the development in micro fabrication technology, MN configurations of biocompatible materials are typically achieved using relatively simple methodologies such as mold casting [34–37] and drawing lithography [38, 39]. However, these methods are not suitable for conductive materials including metals compared to removal machining including MEMS processes. Furthermore, an electrochemical interface should be realized at the interface between MN-based electrodes and biological fluids. For this reason, many studies focus on the coating of MNs with conductive materials represented by Au, Pt, and Ag/AgCl as the WEs, counter electrodes (CEs), and reference electrodes (REs), respectively, in order to enable an MN to function as an electrochemical electrode. In this methodology, metal [40–46], epoxy [47–49], SU-8 [50–53], and polymer [53–59] MNs are coated with metal layers using electroplating or vapor deposition. The covering metal layers are connected to the extract electrodes and the measurement system to conduct the input and output signals.

**Fig. 1:**
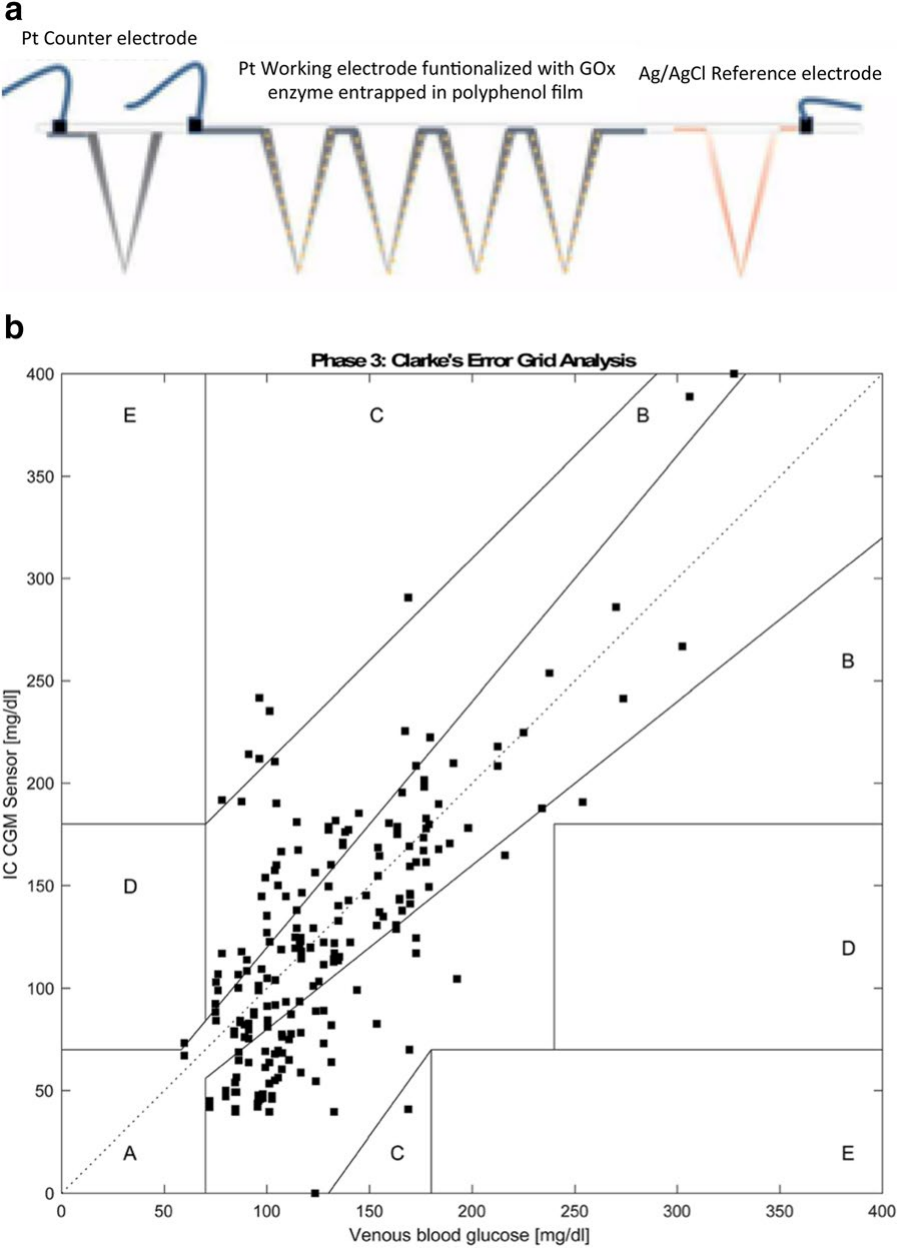
**a** Schematic of the coated MN-based electrodes, **b** Measurement of Clarke error in the 24 h clinical test of the glucose monitoring system with the MN array (Reproduced with permission [52]. Copyright 2018, the Royal Society of Chemistry)

MNs coated with conductive materials to be used as glucose sensors have in been realized mainly by using the immobilized GO_x_ on the surface of the MN [44, 46, 51–53, 55–58, 60]. The Cass’s group developed the polycarbonate [60] and epoxy-based MN array [52] coated with Pt as WE and CE, and Ag/AgCl as the reference electrodes for CGMS application. The MN array was designed with three separate electrode areas and fabricated by the mold casting method, as shown in Fig. 1a. This coated MN-based glucose sensor is developed with the measuring setup enabling long-term glucose monitoring in a mobile and wearable system. The polycarbonate-based MN array was evaluated for its performance in Type 1 diabetes patients for 24 h after conducting safety tests on healthy human bodies for 6 and 24 h. The result shows the feasible accuracy of the glucose monitoring for Type 1 diabetes, as described in Fig. 1b.

### MNs integrated with electrodes

Another approach to devise an MN as a sensing probe is to integrate a MN-based mechanical guide with the micro-sized electrodes. The non-MN shaped electrodes are integrated with the MNs by being enclosed in the hollow MNs [61–66]. In this method, the hollow MNs work as mechanical guides for the enclosed conductor (e.g. carbon paste) to puncture the skin and support the electrodes inside the MNs because, as mentioned above, the electrode materials are not suitable for the MN fabrication by a simple process. This approach provides the advantage that the immobilized enzyme and the electrodes are protected by the surrounding hollow structure of MNs during the penetration into the skin and in the measurement process.

On the contrary to the hollow MNs enclosing conductive components, a few studies focused on the fabrication of electrodes on in-plane probes, which is obtained from a Si wafer by CMOS and MEMS-based process [66, 67]. These MN-shaped sensing probes can be likened to conventional sensing probes for neural signal measurement [68–71]. In this approach, gold or platinum electrodes with circuits and contact pads are fabricated on in-plane micro-sized probes, which are provided a configuration of MNs or assembled with hollow MNs. Although this concept leads to a lesser number of MNs compared to out-of-plane MN arrays in accordance with the fabrication principle, CMOS-based probes are relatively suitable for integration with the setup of the measurement system compared to other MN-based sensors.

**Fig. 2:**
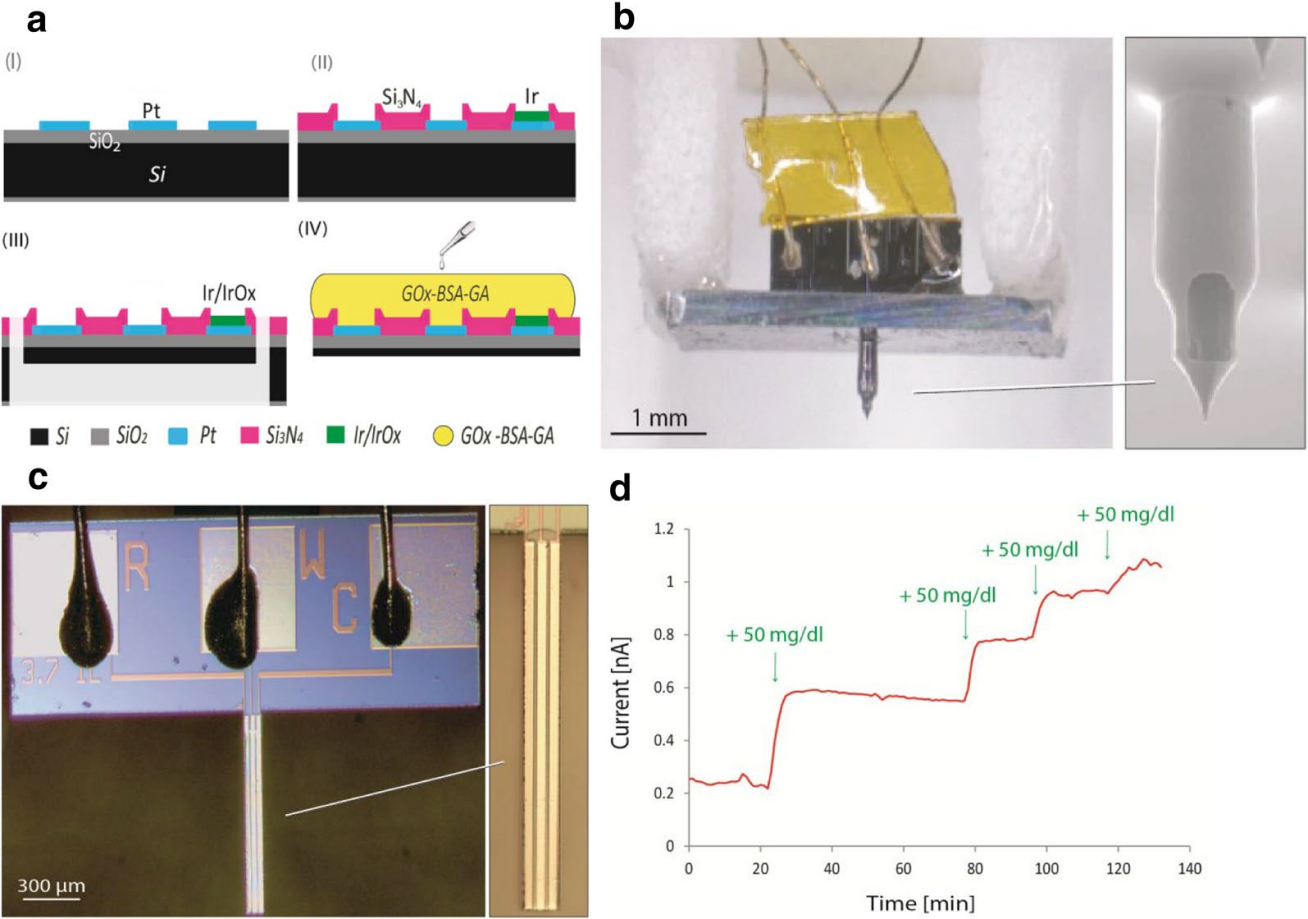
**a** Cross-sectional fabrication process of the electrodes on the Si MN. **b** An optical image of the assembled hollow MN and measurement setups. SEM image of the hollow MN is shown on right hand, **c** A micrograph of the fabricated electrodes on the Si probe, **d** Amperometric measurement of glucose by the hollow MN-based sensor (Reproduced with permission [66]. Copyright 2018, IEEE)

 Ribet et al. reported the fabrication of a Si-based hollow MN with side openings that encloses the platinum WE and CE and the iridium oxide RE on a micro-sized probe fabricated by the CMOS processes (Fig. 2a) [66]. In this approach, ISF permeates the side openings and is sequentially measured by the three electrodes enclosed in the hollow MN, as illustrated in Fig. 2b, c. The device successfully detects the amperometric signal according to the given glucose concentration even though only short time measurement is performed, as shown in Fig. 2d.

## MNs as a biological fluid collectors

### Hollow MNs

An alternative way to access biological information is to extract body fluids through the micro pores punctured by the MNs. In this category, the MNs are defined as biological fluid collectors creating a transdermal fluidic path at the interface between the inner tissue including the dermis and the outer part of the skin. The MNs collecting biological fluids such as blood and ISF have attracted the interest of researchers as they have the potential to be utilized in the existing ex vivo diagnostic systems including pTAS devices. However, it is necessary for the MN-based collector to collect at least a few pL of liquid for a reliable measurement of the analytes [72–74], whereas the MN-based sensing probes have a high availability of biological fluids under the surface of the skin. Therefore, the sampling performance is critical to the MN-based fluid collectors.

Research on the application of hollow MNs for the collection of biological fluid was initiated earlier than that of other types of MNs, probably due to their suitability to the sampling of a sufficient amount of the biological fluid in a manner similar to conventional hypodermic needles. The hollow MNs extract ISF or blood into their bores by a capillary action or external negative pressure. As the hollow MNs were put to use as micro injection needles for localized drug delivery into the skin at the initial stage of the development of research in MNs [3, 75], the methods of fabrication and theoretical analysis of the hollow MNs are well investigated [4, 76–81]. As a result, the hollow MNs for fluid extraction are mainly made of Si or metals using MEMS-based micromachining technologies, including chemical etching or electroplating. In addition, bores in hollow MNs need to be precisely aligned on inlets of fluidic measurement systems to collect biological fluids for a rapid analysis. According to this strategy, hollow MN-based sensing systems are highly developed for the measurement of protein [82], ions [83], glucose [84–90], and other analytes [91–93] in either blood or ISF.

**Fig. 3:**
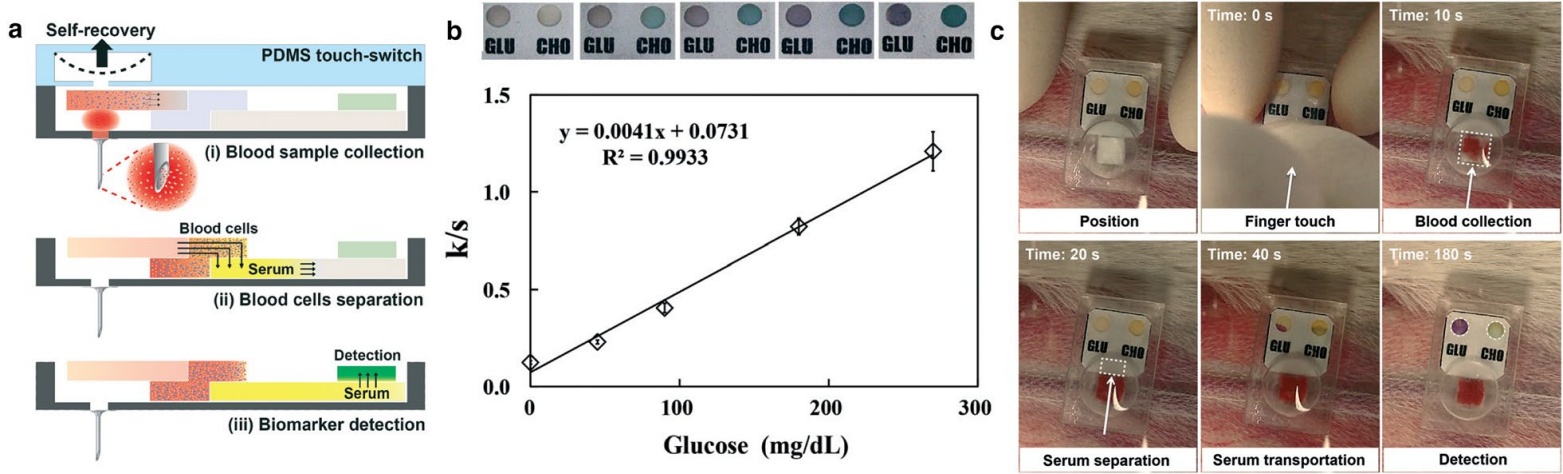
**a** The sampling and measurement process of blood using a hollow MN and paper-based sensor. **b** The change of color (above) and calibration curves for glucose measurement (below). **c** A series of optical images of the blood sampling and glucose measurement in a rabbit ear with the integrated hollow MN and the paper-based sensor (Reproduced with permission [89]. Copyright 2015, the Royal Society of Chemistry)

 The Jung’s group presented a vacuum-pressure aided blood extraction system with Ni hollow MN for a colorimetric glucose sensor [89]. They connected the hollow MN fabricated by electroplating to a PDMS micro chamber, which is elastically deformable by a finger push to evacuate the air inside, as illustrated in Fig. 3a. The manually generated negative pressure drives the blood from the vessel into the chamber via the hollow MN, followed by a capillary transport of the blood through a filtering and GO_x_-immobilized paper channel. The glucose level is observed by comparing the colorimetric change of the paper (Fig. 3b), which results in a blood glucose measurement of a rabbit ear (Fig. 3c). In terms of point-of-care glucose sensors, the hollow-MN based measurement system showed the potential to be applied for clinical use as a miniaturized system of the present glucose monitoring kit using finger pricks and colorimetric reactions.

### Swelling MNs

An alternative method to the collection of ISF by using an MN array is to employ swelling materials for the body for the MN. It has been demonstrated that hydrogel MNs are capable to extract ISF by swelling, which results in a fluidic conduit [94–97], as well as to release the drug contained in the MN matrix at the same time [98–101].

ISF is absorbed in the dry hydrogel MNs by diffusion, after their penetration into the skin, which has to be followed by the process of separation of the sampled ISF from the MN array, by using centrifugation, either with or without immersion into a solvent. Although the swelling MNs intrinsically need the additional process to remove the analytes, the main fabrication process by mold casting is relatively simple and they have the ability to collect ISF efficiently due to the relatively large capacity of the sampling volume. This approach is also applied to the measurement of glucose levels [96, 97].

### Porous MNs

Although the porous MNs were widely considered as drug delivery devices [34, 102–106], the porous MN array as an ISF collector has only been developed in recent years [107–110]. Porous MNs have the capability to absorb ISF by a capillary action depending on the geometry of pores in MN bodies and their hydrophilicities. Although the concept of ISF extraction by the porous MNs has been proposed before, conventional porous MNs need to be centrifuged or dipped in a solvent to separate the absorbed ISF, in the same manner as in the case of the swelled MNs, which leads to an increase in the number of steps involved in analysis.

**Fig. 4:**
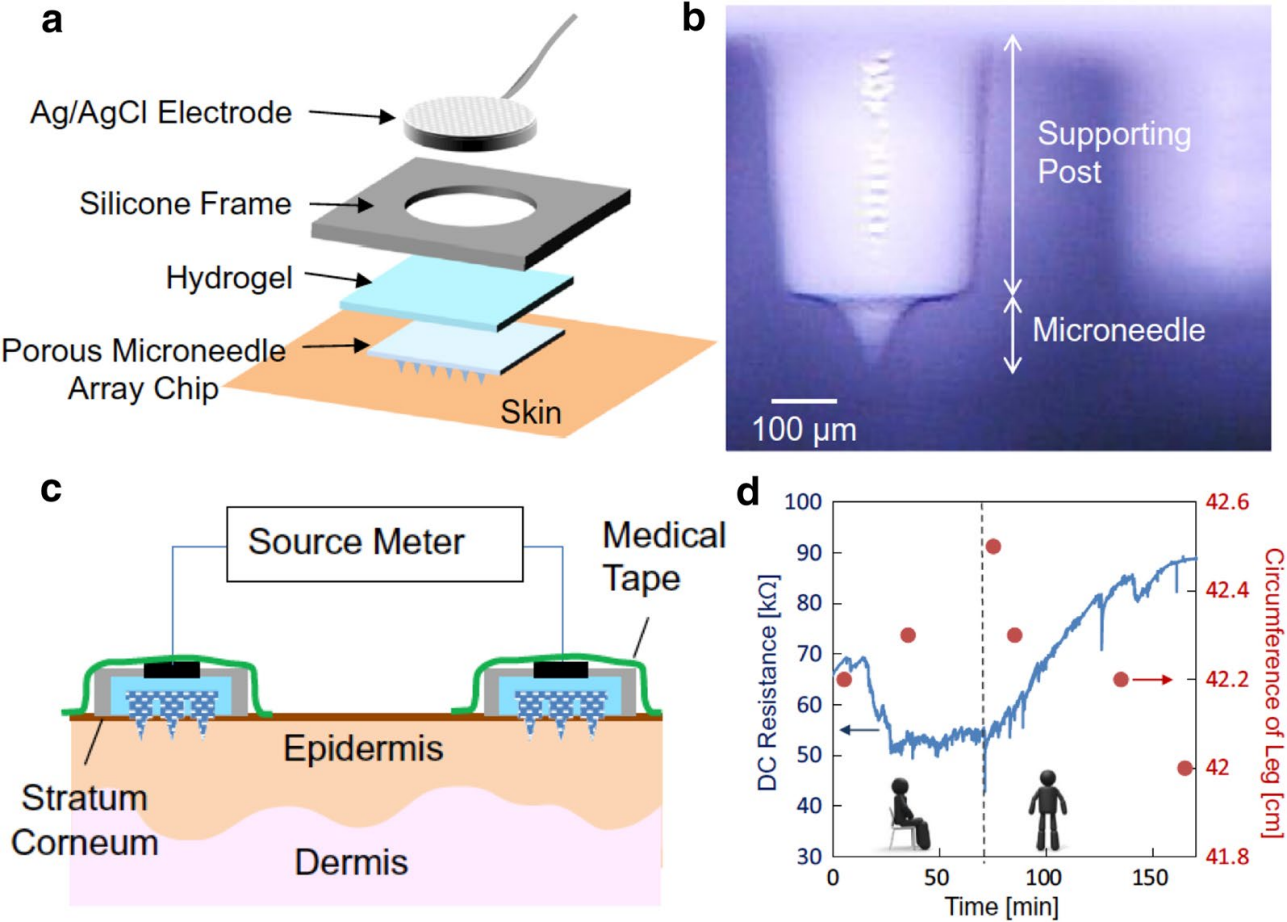
**a** Schematic of the porous MN-based intercellular swelling measurement system. **b** A micrograph of the porous PEG MN. **c** Schematic of the DC resistance measurement by two porous MN array. **d** The DC resistance measured by the porous MN array and the circumference changed by a movement (Reproduced with permission [110]. Copyright 2015, Springer Science + Business Media, LLC)

The first successful study of a porous MN array for ISF collection and a fluidic connection to a external measurement system was reported by the Nishizawa’s group [109, 110]. As the efficiency of collection by the capillary action into the porous MNs is rated lower than that by the hollow MNs [111], the present study employs the porous MN array only as a transdermal fluidic path and not for the direct outward flow of ISF, as illustrated in Fig. 4a. The absorbed ISF in the polyethylene glycol (PEG) MN array (Fig. 4b) was fluidically connected to a hydrogel located on the rear side of the MN array, so that the electrolytes conduct current from electrodes to the dermis through the porous structure. Figure 4c describes the schematic of the measurement of the DC electric resistance on human skin, whose results showed the detection of an intercellular swelling according to the change of distance between two porous MN array on a leg skin, as illustrated in Fig. 4d. Even though the target of the measurement was not glucose but electrolytes in ISF in this report, the transdermal fluidic path created by the porous MN array made of biodegradable materials such as PEG is very promising for CGMS. In addition to this approach, Takeuchi et al. reported the fluidic connection between a porous structure and a microfluidic channel network fabricated by a MEMS process [112].

## Towards CGMS using MNs

Considering the research history of the micro-sized glucose sensors, MNs functionalized for bio-sensing systems are comparatively recent. Hence, there are still challenges to be faced in the development of MN-based bio-sensing devices, especially for CGMS.

As discussed above, the MN-based electrodes, especially MNs coated with conductive layers, have been successfully indicated to monitor the blood glucose level continuously. This is because the MN-based electrodes are in direct contact with a sufficient amount of fluids beneath the skin for a reliable measurement of glucose, in a similar manner to conventional implantable sensors. However, from the perspective of the application of CGMS devices, the MN-based electrodes have certain drawbacks. One is a common problem of implantable sensors, that is, the loss of the sensitivity owing to surface fouling and enzyme inactivation induced by proteins and immune response [113–115]. This can be an obstacle in the use of a CGMS device in the current situation, as a longer period is required for glucose monitoring as commercially available devices achieving up to 14 days of monitoring of blood glucose [21]. Additionally, measurement methodologies of biological analytes, in the use of MNs as in vivo sensing probes, are basically limited to the enzymatic electrochemical measurement. This is because such MN-based electrode sensors are compatible not with complicated measurement systems but with relatively simple sensing principle such as electrochemical reactions at the surface of MNs. The limited measurement methodology, using only electrochemical sensing with conventional electrodes, restricts the use of more accurate methods of measurements such as FET-based sensors [116–118], which results in a limited number of measurable biomarkers.

On the other hand, the use of MNs as biological fluid collectors have an advantage over their use as sensing probes because of the developed technology of ex vivo analysis of extracted biological samples. In particular, pTAS devices meet the purpose of the MN-based fluid collector by achieving a small amount of direct flow of ISF or blood from inside the skin to measurement systems. From this perspective, the hydrogel-forming swellable MNs have a disadvantage of the requirement of an additional step to separate the absorbed ISF from the MN array. As microfluidic CGMS devices composed of microchannels have already been proposed using infrared light [119], viscosity-sensitive cantilever [120, 121], and enzymatic electrochemical measurement [122–124], the integration of the fluid collecting MNs with these devices would substantiate minimally invasive CGMS devices. However, the fabrication of such MNs is relatively complicated because, in order to integrate a MN array as a fluid collector with CGMS devices, fluidic channels in MNs should be precisely connected to the microchannels in the device. Furthermore, the sampling rate of MNs is crucial for CGMS. As reported by Samant and Prausnitz, the rank order of the amount of ISF collected by MNs is as follows: hydrogel MN (0.0030 pL per MN (12 h insertion)) < paper-based porous MN (0.0033 pL per MN (20 min insertion)) < hollow MN (0.01–0.03 pL per MN (20 min insertion)) [111]. According to this analysis, hollow MNs enable sufficient extraction of ISF while the porous MN sampling rate is limited by a diffusion through the dermis. However, the porous MN provides advantages such as its applicability for biodegradable materials including PEG and the simple fabrication and assembly with microfluidic systems, whereas the hollow MN must be made of non-biocompatible materials and fabricated by a complex process which limits the microfluidic system design.

## Conclusion

In this paper, the applicabilities of functionalized MNs to CGMS were discussed. The MN-based in vivo sensors have been applied to diagnostic systems even in a clinical experiment level, by being functionalized as electrodes, especially for glucose measurement. However, the MNs as sensing probes have been facing the challenges similar to those of the implantable sensors, such as the impaired sensitivity and the limited measurement methodology. On the other hand, the MN-based trans-dermal fluidic channels can be highlighted with the potential to be combined with recently developed pTAS technologies. Therefore, the use of MNs as biological fluid collectors should be investigated for integration with the existing ex vivo diagnostic systems and for a more efficient sampling mechanism of ISF. The development of functionalized MNs in those ways will contribute to the realization of commercially available CGMS devices in a minimally invasive manner instead of conventional glucose monitoring devices with pain.
