# Correction to: Functionalized microneedles for continuous glucose monitoring

**DOI:** 10.1186/s40580-018-0169-7

**Published:** 2018-11-29

**Authors:** Kai Takeuchi, Beomjoon Kim

**Affiliations:** Institute of Industrial Science, The University of Tokyo, 4‑6‑1 Komaba, Meguro‑ku, Tokyo, 153‑8505 Japan

## Correction to: Nano Convergence (2018) 5:28 10.1186/s40580-018-0161-2

After publication of this article [1], the author noticed there are some conversion errors with the below characters and units in the article body text. The corrections are detailed below:

1. ‘pTAS’ should be ‘µTAS’
2. ‘pm’ should be ‘µm’

We apologize for the inconvenience caused.
